# Lightweight Magnetic Nanoparticle–Graphite Composites with Tunable and Enhanced Electromagnetic Wave Absorption Properties

**DOI:** 10.1021/acsomega.5c13545

**Published:** 2026-04-21

**Authors:** Dilsad Dolunay Eslek Koyuncu, Nail Bugra Kilic, Huseyin Cagri Yavuz, Sude Nur Demiran, Huseyin Bozkurt, Fatima Nur Cubukcuoglu, Rukiye Ozdemir

**Affiliations:** † 37511 Gazi University, Faculty of Engineering, Chemical Engineering Department, Ankara 06570, Turkey; ‡ Research and Technology Centers, Turkish Aerospace, Ankara 06980, Turkey

## Abstract

In this study, Fe, Ni–Fe, and Co–Fe magnetic nanoparticle–graphite hybrid composites are synthesized via the coprecipitation method. The effects of metal type, metal/C mass ratio, metal salts (anhydrous or hydrated Fe salts), and pH adjustment agents (NH_4_OH or NaOH) on product properties and electromagnetic interference (EMI) shielding efficiency are examined. X-ray diffraction (XRD) confirms the formation of a cubic spinel structure. Fourier transform infrared spectroscopy (FT-IR) identifies the functional groups in the composites. Scanning electron microscopy (SEM) images show that increasing the Fe ratio from 1 to 8 leads to the increase in particle size from 19 to 27 nm in average, while the choice of pH adjustment agent has a minimal effect on morphology. The use of anhydrous metal salts results in larger and more porous particles. Vibrating Sample Magnetometry (VSM) analysis reveals that the FeG6.14Na sample exhibits the highest saturation magnetization (59.8 emu/g), while the NiFeG6.14Na sample, having smaller particle size, shows the lowest (29.6 emu/g). X-ray photoelectron spectroscopy (XPS) confirms the coexistence of Fe^2+^, Fe^3+^, Ni^2+^, and Co^2+^ oxidation states. The band gap of the samples ranged from 1.18 (CoFeG6.14Na) to 1.65 eV (FeG4NaS). EMI shielding tests indicate that Fe-containing composites achieve better wave absorption performance than NiFeG6.14Na (1.57%) and CoFeG6.14Na (1.81%). Overall, Fe-based composites demonstrate superior magnetic and EMI shielding properties, making them more effective for absorption-based applications.

## Graphical abstract

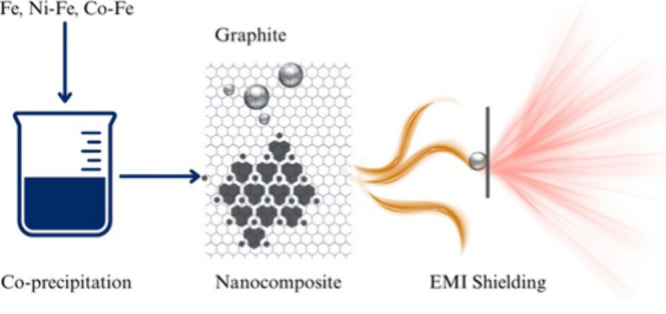

## 1 Introduction

The rapid proliferation of electronic devices and the advancement of wireless communication technologies have led to a significant increase in environmental electromagnetic radiation. Electromagnetic interference (EMI) is defined as unwanted electromagnetic signals generated by such radiation that adversely affect the performance of electronic systems. EMI usually occurs in two main ways: transmitted (conducted) and radiated (radiated) interference. The transmitted EMI is emitted through electrical conductors, while the emitted EMI is emitted in the form of electromagnetic waves in free space. These types of interference can have different effects depending on the sensitivity of electronic devices and their operating principles.

EMI can cause signal distortions, data loss, malfunction, or even complete failure of electronic equipment. Particularly in critical sectors such as medical devices, aerospace, defense, and telecommunications, EMI-induced problems can jeopardize device reliability and human safety. Moreover, the potential adverse health effects of electromagnetic pollution have become an increasing concern within both public and scientific communities.^1^ Therefore, controlling electromagnetic waves and mitigating their harmful impacts have gained vital importance alongside technological progress. One of the fundamental methods employed for EMI protection is electromagnetic shielding.

EMI Shielding involves using a barrier that reduces the impact of unwanted electromagnetic fields on electronic devices. Electromagnetic shielding is of great importance in terms of protecting electronic devices and living beings from the negative effects of electromagnetic waves with the spread of modern technology. While this protection prevents sensitive electronic devices from being affected by electromagnetic interference, it also helps to protect human health from potential damage from electromagnetic pollution. EMI shielding is widely used in industries like aerospace, military, medical, and consumer electronics. There are three main mechanisms in electromagnetic interference protection. These are reflection, absorption, and multiple reflection (Figure 1). Reflection is the reflection of EMI waves back from the surface of the material, absorption is the weakening of EMI waves as they enter the material, absorbing their energy, and multiple reflection is the reflection, weakening and becoming ineffective within the material. Electrically and magnetically conductive materials increase the absorption mechanism. Multiple Reflection usually occurs with composite materials having large surface areas and porous structures.

**Figure 1:**
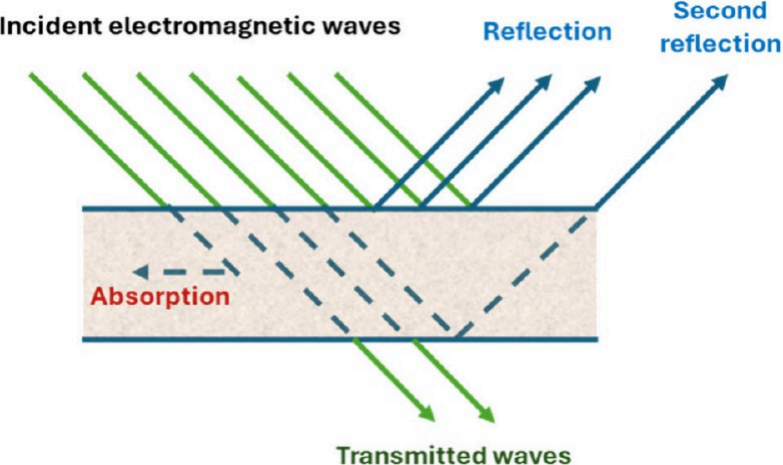
Mechanism of EMI shielding.

Shielding effectiveness is defined as the ratio of incident energy to the remaining residual energy after interaction with a shield. When an electromagnetic wave encounters a shielding material, both absorption and reflection mechanisms contribute to its attenuation. The residual energy refers to the portion of the wave that is neither reflected nor absorbed by the shield but instead emerges from the other side (Figure 1).^2^

Therefore, the research and development of shielding activities of different materials and structures are becoming more and more important with the advancement of technology. Shielding involves using materials or structures that reflect, absorb, and attenuate electromagnetic waves, thereby safeguarding devices and environments.^3^ The shielding effectiveness depends on multiple factors, including the electrical conductivity, magnetic permeability, dielectric properties, thickness, structural characteristics, and surface morphology of the shielding material.^4^ Materials with high electrical conductivity predominantly protect by reflecting electromagnetic waves, while those with strong magnetic properties enhance absorption. Multilayered shielding architectures can further boost effectiveness through multiple reflection mechanisms.^1^

Historically, metal-based materials have been the most widely used for EMI shielding, owing to their excellent electrical conductivity. Metals can absorb, reflect, and transmit EMI due to their high electrical conductivity. In contrast, insulating materials such as rubber and plastics are permeable to EMI.^5^ The fact that metals provide both electrical and thermal conductivity makes them indispensable in many applications. In this context, metal-based hybrid materials are emerging as an important alternative for EMI shielding.^3^ However, metals exhibit limitations such as high density, susceptibility to corrosion, manufacturing difficulties, and lack of flexibility, which restrict their application in modern lightweight and flexible electronics.^5^ Metal-based hybrid materials enhance EMI shielding performance by combining the conductive and magnetic properties of metals with magnetic components such as graphite, iron salts cobalt, and nickel. At the same time, they offer advantages such as low weight, flexibility, corrosion resistance, and ease of manufacturing, making them a more efficient option compared to traditional metal solutions. These materials have made significant progress in meeting the EMI shielding requirements of evolving electronic devices and systems.^4^ Recently, research has shifted toward polymer composites, **c**eramics, and carbon-based nanomaterials, which offer tunable electrical and magnetic properties, corrosion resistance, and low weight.^3^ By combining these materials in hybrid structures, advanced electromagnetic shielding materials can be developed. Traditional polymers are nonconductive, but metal-coated polymer fibers offer better shielding than uncoated carbon fibers. Naturally conductive polymers, such as polythiophene and polyaniline, are useful for shielding due to their conductivity, though they have lower mechanical strength and higher costs. These polymers are used as matrix materials in composites to improve electrical continuity.^6^ Cement-based materials provide durable and effective solutions for electromagnetic interference (EMI) shielding. Although primarily used in construction, they can also be applied in environments requiring electromagnetic protection. Cement is often combined with ferrites, carbon, metal oxides, and other shielding materials to enhance EMI protection.^7^ Ceramics are not as commonly used in electromagnetic interference (EMI) protection as metals or carbon-based materials. The main reason for this is that ceramics have relatively low conductivity. However, among ceramics, metal carbides (such as silicon carbide and titanium carbide) are relatively more attractive options due to their partial conductivity.^3^

Recently, hybrid composites combining carbon-based materials with magnetic metal nanoparticles such as iron, nickel, and cobalt have emerged as promising candidates that simultaneously leverage electrical conductivity and magnetic loss mechanisms to achieve enhanced shielding performance.^8^ These hybrid materials improve electromagnetic wave absorption, enabling optimized attenuation while maintaining lightweight, thin, flexible, and corrosion-resistant properties suitable for broader applications.^9^ Nevertheless, challenges remain in synthesizing, compositional optimization, microstructural control, and performance evaluation of these composites, which continue to be active areas of research.

Carbon nanotubes (CNTs), graphene, graphene oxide, carbon fibers, and carbon black have demonstrated significant advantages in EMI shielding applications due to their superior electrical conductivity, large surface area, and mechanical flexibility.^10^ Carbon nanofibers (CNFs) and nanotubes have been increasingly used as substitutes for short carbon fibers (SCFs) due to their higher aspect ratio and electrical conductivity, They enhance electromagnetic losses even at low filler content while reducing the material’s thickness and weight.

Advanced characterization techniques play a critical role in developing EMI shielding materials. Methods such as Fourier transform infrared spectroscopy (FTIR), X-ray diffraction (XRD), scanning electron microscopy (SEM), vibrating sample magnetometer (VSM), and X-ray photoelectron spectroscopy (XPS) provide detailed insights into the chemical composition, crystallinity, morphology, and magnetic properties of synthesized materials.^3^ These insights guide material design and enable optimization to maximize shielding effectiveness.

As a result, it was concluded that carbon-based nanoparticles are frequently used in EMI shielding applications. Properties such as lightness, functionality, thermal stability, low cost, and processability are the features that are at the forefront of their preference in these applications.^10^ In the use of carbon-based materials, derivatives such as graphene, graphene oxide, carbon nanotube, graphite, carbon fiber, and carbon black attracted attention. At the same time, it was evaluated that hybrid composites formed with magnetic metal (Aq, Fe, Ni) nanoparticles increased shielding efficiency.^8,9^

In this study, magnetic nanoparticle–graphite hybrid composites were synthesized, and characterized, and their electromagnetic shielding performances were comprehensively evaluated. The effects of varying metal-to-carbon ratios, synthesis conditions, and metal species on shielding efficiency were systematically investigated. The results contribute to the development of high-performance, lightweight, and thin EMI shielding materials. This work serves as a significant reference in advancing and optimizing materials for protecting electronic devices against EMI.

## 2 Experimental Studies

The main purpose of this study is the production and application of magnetic nanoparticles optimized for electromagnetic absorption within the scope of electromagnetic shielding applications. In this context, the experimental method is given.

### 2.1 Materials

Iron­(II) chloride tetrahydrate (FeCl_2_.4H_2_O), iron­(III) chloride hexahydrate (FeCl_3_.6H_2_O), iron­(II) chloride (FeCl_2_), iron­(III) chloride (FeCl_3_), ammonium hydroxide (NH_4_OH), sodium hydroxide (NaOH), nickel­(II) chloride tetrahydrate (NiCl_2_.6H_2_O), cobalt­(II) chloride tetrahydrate (CoCl_2_.6H_2_O), graphite, hydrochloric acid (HCl), distilled water.

### 2.2 Synthesis of Magnetic Nanoparticle–Graphite Composite Samples

The required amounts of metal salts (Fe, Ni–Fe, Co–Fe salts) were placed in a volumetric flask and mixed with distilled water (150 mL), and stirring continued until the salts were completely dissolved. After dissolution, the required amount of graphite was added to the solution. The temperature was then set to 85 °C. Once the solution reached 85 °C, NaOH (or NH_4_OH) (13.5 mL) was added to adjust the pH to 10. In the literature, pH adjustment is done with NH_4_OH. In this study, dilute NaOH was used for ease of application. After the pH adjustment, a reflux system was attached to the flask, and the mixture was kept under these conditions for an hour. The aim of this setup is to avoid solvent loss by minimizing evaporation.

After mixing at 85 °C, the solution was allowed to cool down to room temperature. At this stage, the solid content in the solution was expected to precipitate according to the following reactions (Reaction [Disp-formula eqR1] and Reaction [Disp-formula eqR2]).

R1 $${Fe}_{\left(aq\right)}^{2+}+{2Fe}_{\left(aq\right)}^{3+}+{8OH}_{\left(aq\right)}^{-}\to {Fe}_{3}O_{4\left(s\right)}\text{↓}+{4H}_{2}O_{\left(l\right)}$$

R2 $$M_{\left(aq\right)}^{2+}+{2Fe}_{\left(aq\right)}^{3+}+{8OH}_{\left(aq\right)}^{-}\to {MFe}_{2}O_{4\left(s\right)}\text{↓}+{4H}_{2}O_{\left(l\right)},\left(M:\mathrm{Ni},\mathrm{Co}\right)$$

The solid part of the solution was collected and transferred into a centrifuge tube. The centrifugation process was performed at 20 rpm for 5 min. This step was repeated until the liquid was completely separated from the suspension. The sample taken from the centrifuge was dried in an oven at 90 °C for 24 h. In this study, the effect of different metals (Fe, Ni–Fe, Co–Fe), metal salts (anhydrous or hydrated Fe salts), pH adjustment agents (NH_4_OH or NaOH), and metal/C mass ratio (*n:* 1, 4, 6.14, 8) on the properties of magnetic nanoparticle–graphite composite were investigated. Table 1 shows the details of the samples prepared. In this study, the sample notation is “xFeGnz”. In this nomenclature, x represents Ni or Co metal (if any), Fe represents iron, G represents graphite, n represents the total metal/C ratio, and z represents the pH adjustment agent (N: NH_4_OH, Na: NaOH) used in the synthesis. For the samples labeled with the letter ″S,″ anhydrous forms of the iron salts (FeCl_2_ and FeCl_3_) were used instead of their hydrated forms. In all samples, FeCl_2_.4H_2_O and FeCl_3_.6H_2_O metal salts were used as iron salts in a molar ratio of Fe^2+^/Fe^3+^=0.61. For example, in the CoFeG6.14Na sample, “Co” and “Fe” represent the presence of cobalt and iron metals, “G” represents graphite, “6.14” represents the mass ratio of (Fe+Co) to graphite, and “Na” represents NaOH as the pH adjusting agent.

**Table 1:** Samples and Synthesis Conditions

Samples	Metal/C (by mass %)	pH agent	Fe Salts
FeG1NaS	1	NaOH	FeCl_2_, FeCl_3_
FeG1Na	1	NaOH	FeCl_2_.4H_2_O, FeCl_3_.6H_2_O
FeG4Na	4	NaOH	FeCl_2_.4H_2_O, FeCl_3_.6H_2_O
FeG8Na	8	NaOH	FeCl_2_.4H_2_O, FeCl_3_.6H_2_O
FeG4NS	4	NH_4_OH	FeCl_2_, FeCl_3_
FeG4NaS	4	NaOH	FeCl_2_, FeCl_3_
FeG6.14Na	6.14	NaOH	FeCl_2_.4H_2_O, FeCl_3_.6H_2_O
NiFeG6.14Na	6.14	NaOH	FeCl_2_.4H_2_O, FeCl_3_.6H_2_O
CoFeG6.14Na	6.14	NaOH	FeCl_2_.4H_2_O, FeCl_3_.6H_2_O

### 2.3 Characterization of the Composite Samples

The crystalline phases of the synthesized samples were characterized by XRD analysis, conducted using a Rigaku Ultima IV diffractometer equipped with a Cu Kα radiation source *(λ* = 0.15406 nm) operated at 40 kV and 30 mA. The functional and structural groups present in the samples were identified using FT-IR spectroscopy coupled with an Attenuated Total Reflectance (ATR) accessory. The measurements were conducted in the mid-infrared region (4000–400 cm^–1^) at a spectral resolution of 4 cm^–1^ using a Jasco 4700 ATR/FT-IR spectrophotometer. The surface chemical composition, metal ratio, and states of the metals were examined using XPS analysis, carried out with a PHI 5000 VersaProbe. The surface morphologies and chemical composition of the composites were determined by SEM and EDS analysis using QUANTA 400 F Field Emission Scanning Electron Microscope (FE-SEM) equipment. Transmission electron microscopy (TEM) images of the sample were monitored using Tecnai G2 F30 Electron Microscope device. The magnetic properties of the samples were determined by VSM analysis. VSM analyses were conducted using a Lake Shore 7407 model magnetometer device. Surface areas of the samples were measured by Anton Paar Nova 600 device after degassing for 3 h at 120 °C. UV–vis DRS spectra of the materials were determined using a PerkinElmer Lambda 35 UV–vis spectrophotometer in the 200–1100 nm range with a resolution of 1 nm. Prior to analysis, samples were mixed with KBr, the reflectance standard material, formed into pellets, and their moisture was removed.

### 2.4 Determination of the EMI Shielding Effectiveness

#### 2.4.1 Molding the Samples with Paraffin

The synthesized materials were intended to be mixed with paraffin at a specific ratio. A measured amount of the synthesized material and paraffin containing 4% oil was weighed to achieve the desired mass ratio in the final sample.

For molding, the paraffin was placed into a beaker and melted in a water bath maintained at 60 °C. Once fully melted, the synthesized material was added to the beaker. The mixture was then subjected to ultrasonic treatment for 2 min to ensure proper homogenization, which is critical for achieving a uniform material before molding. Since paraffin transitions from a liquid to a solid phase at room temperature, the mixture needs to be poured into molds quickly to prevent premature solidification. In this study, molds with dimensions of 22.86 mm × 10.16 mm. were used.

#### 2.4.2 Electromagnetic Shielding Tests

Electromagnetic tests were performed using the waveguide method defined in ASTM D 5568 standard. The samples were prepared to be placed in a WR 90 standard waveguide, and the transmission and reflection values of electromagnetic waves in the frequency range of 8.2–12.4 GHz were measured using a Rohde and Schwarz vector network analyzer (VNA). The dimension of the sample, which is 22.86 mm × 10.16 mm, was selected for the waveguide test frequency range. During the measurements, the sample was tightly placed inside the waveguide to ensure minimal air gaps and to prevent signal leakage around the edges. Electromagnetic waves were measured via the waveguide using the VNA connected to the input and output ports of the waveguide via waveguide-to-coaxial adapters. To ensure the reliability of the results, each measurement was repeated at least three times, and the average results were reported.

## 3 Results and Discussion

### 3.1 XRD Results

In this study, the effect of different metals on the properties of magnetic nanoparticle-containing composite samples was investigated using Fe, Ni–Fe, and Co–Fe metals. The primary objective was to compare the influence of different metal substitutions (Fe, Ni, Co) on the spinel ferrite structure and to evaluate how these variations affect crystallite size and structural integrity. Figure 2 shows the XRD patterns of the FeG4Na, NiFeG6.14Na, and CoFeG6.14Na samples along with the corresponding Bragg reflection positions. To compare effectively, in all samples, the molar ratio of Fe to C was maintained at 4. While the total metal/C mass ratio was 6.14 in the NiFeG6.14Na and CoFeG6.14Na samples, it was 4 in the FeG4Na samples.

**Figure 2:**
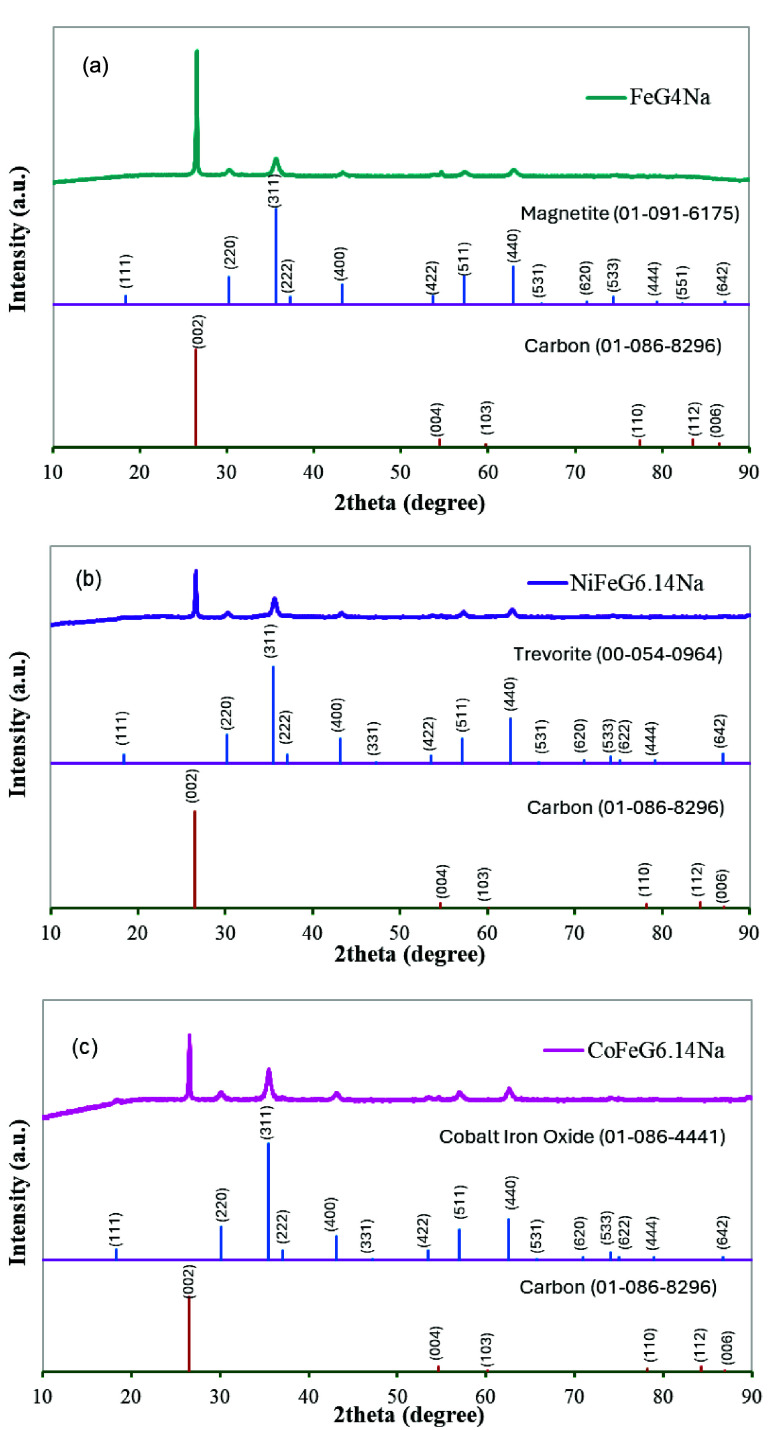
XRD patterns of (a) FeG4Na, (b) NiFeG6.14Na, and (c) CoFeG6.14Na samples.

According to the literature, graphite exists in both hexagonal and rhombohedral crystal phases. The hexagonal phase is characterized by four distinct diffraction peaks observed at 2θ values of 26°, 44°, 55°, and 77°, corresponding to the (002), (100), (004), and (110) crystallographic planes, respectively.^11^ In all samples, diffraction peaks corresponding to graphite were present with varying intensity levels. A main graphite peak appeared at around 2θ ≈ 26.5°, and among all samples, FeG4Na showed the highest intensity for this reflection. This may be due to the lower total metal ratio of the FeG4Na sample. Crystallite sizes of the graphite phase in these samples were calculated using the Scherrer Equation. Crystallite sizes of the graphite phases in the FeG4Na, NiFeG6.14Na, and CoFeG6.14Na samples were calculated as 37.2, 34.3, and 28.1 nm, respectively.

In the XRD patterns of the FeG4Na sample (Figure 2-a), distinct diffraction peaks were observed at 2θ ≈ 30.19°, 35.60°, 43.26°, 57.24°, and 62.87°. These peaks corresponded to the (220), (311), (400), (511), and (440) planes, respectively, and represented the Fe_3_O_4_ (magnetite) crystal phase in the cubic spinel structure.^12^ In particular, the intense peak corresponding to the plane at 35.6° with an interlayer distance of 2.5199 Å revealed that the magnetite structure was dominant. At the same time, observations of the other planes show that the crystalline phase was formed in a pure and ordered manner.^13^ The Rietveld refinement applied to the XRD pattern of the FeG4Na composite sample containing graphite and magnetite (Fe_3_O_4_) showed good level of fit. The fit quality parameters are *Rwp* = 2.62%, *Rp* = 1.80%, *S* = 1.3342 and *χ* ^*2*^ = 1.7800, and although the *χ* ^*2*^ and *S* values are above the ideal “1”, the refinement is still at an acceptable level of accuracy. This showed that the phases were accurately identified, but there might be deviations from the pattern in some regions. As a result of the Rietveld refinement, it was determined that the composite consists of two phases: Fe_3_O_4_ (magnetite) and carbon (graphite). The phase weight ratios were calculated as 27.5% ± 0.4 Fe_3_O_4_ and 72.5% ± 0.4 graphite, respectively. This result showed that the carbon phase is significantly dominant in the composite, and the loading efficiency of Fe_3_O_4_ remains relatively low. The high stability of the graphite phase and the absence of mass loss during synthesis supported this distribution. The Fe_3_O_4_ phase has a typical cubic inverse spinel structure, and the refined lattice parameter a = 8.350 ± 0.002 Å. The calculated unit cell volume was 582.100 Å^3^, which was close to the typical volume of spinel Fe_3_O_4_. The microstrain value was a significant magnitude of 0.71% ± 0.13. This strain level indicates crystal lattice distortions and internal stresses that Fe_3_O_4_ nanoparticles are subjected to while bonding to the graphite surface in the composite structure. This is significant evidence that metal oxide-carbon interface interactions are strong. The refined lattice parameters for the graphite phase were *a = b=*2.4599 ± 0.0008 Å and *c* = 6.709 ± 0.002 Å, confirming the typical hexagonal graphite structure. The calculated unit cell volume for the graphite was 35.161 Å^3^, which is consistent with literature values.^14^ The microstrain in the graphite phase was found to be 0.16 ± 0.04, which is lower than that of the Fe_3_O_4_ phase; this indicates that the graphite layers remain structurally more stable during synthesis.

The XRD patterns of the NiFeG6.14Na sample (Figure 2-b) showed characteristic diffraction peaks at 2θ ≈ 30.22°, 35.61°, 43.32°, 57.19°, and 62.76°. These peaks also corresponded to crystal planes (220), (311), (400), (511) and (440), respectively. The intense peak corresponding to the plane at 35.5° with an interlayer distance of 2.5261 Å related to the structure NiFe_2_O_4_-Trevorite phase. These findings are consistent with literature. Sivakumar et al. (2011) similarly reported prominent and sharp peaks belonging to (220), (311), (400), (511), and (440) planes in the XRD pattern of pure NiFe_2_O_4_ nanoparticles synthesized by poly­(vinyl alcohol) (PVA) supported sol–gel autocombustion method. The authors emphasized that these peaks indicate that the crystalline structure was highly formed and a pure spinel phase was obtained.^15^ This structure was also observed in NiFe_2_O_4_/CNT composites synthesized by Fowzia S. Alamro (2024), confirming that the same crystalline phase is formed in this study.^16^ Rietveld refinement performed on XRD data of NiFeG6.14Na sample has high fit quality. The obtained fit indicators were *Rwp* = 1.96%, *Rp* = 1.40%, *S* = 0.9840 and *χ* ^*2*^ *=* 0.9683, showing that the calculated model represented the experimental data quite well. The goodness-of-fit value being very close to “1” supported the reliability of the refined structural and microstructural parameters. Refinement revealed that the composite consists of two main phases: Trevorite (NiFe_2_O_4_) spinel phase and carbon (graphite) phase. Weight fractions were calculated as 38.5% ± 0.8 (Trevorite) and 61.5% ± 0.8 (carbon), respectively. The high proportion of graphite indicated that the spinel phase successfully dispersed on the carbon-based support matrix of the composite. The Trevorite phase has a cubic spinel structure (*a = b=c* = 8.3783 ± 0.0013 Å; *α=β=γ* = 90°) and the calculated unit cell volume was 588.267 Å^3^. These values showed high agreement with the pure NiFe_2_O_4_ lattice parameters reported in the literature and indicate that the spinel structure remains stable in the composite form.^17,18^ The microstrain observed in the Trevorite phase was 0.172% ± 0.012, indicating the presence of slight internal stresses in the crystal lattice due to the synthesis conditions. The carbon phase showed a typical hexagonal graphite structure (*a = b=*2.4431 ± 0.0004 Å, *c* = 6.7138 ± 0.0013 Å; *γ=*120°). The calculated unit cell volume for graphite was 34.705 Å^3^ and a small microstrain value (%0.087 ± 0.017) indicating turbostratic behavior was obtained. The co-occurrence of graphite and spinel phases in the same composite supported the homogeneous distribution of spinel particles on the graphite layers.

In the XRD patterns of the CoFeG6.14Na sample ((Figure 2-c), the peaks at 2θ≈30.10°, 35.47°, 43.09°, 56.98°, and 62.60° pointed to the CoFe_2_O_4_ crystal phase in the spinel structure. The interlayer distance of this phase was 2.5291 Å. It was found that there was a more pronounced distortion in the crystal structure with the effect of Co doping and the peaks appeared with lower intensity. This is in agreement with the study of Kathiravan et al., 2024.^19^ The Rietveld refinement applied to the XRD pattern of the composite CoFeG6.14Na sample containing graphite and cobalt ferrite (CoFe_2_O_4_) showed a high-quality fit. The fit quality parameters were *Rwp* = 1.64%, *Rp* = 1.06%, *S* = 1.0222 and *χ* ^*2*^ *=* 1.0449, indicating that the model reliably represented the experimental data. The closeness of S and *χ* ^*2*^ values to “1” supported the accuracy of the refined structural and microstructural parameters. As a result of the refinement, it was determined that the composite consists of two phases: CoFe_2_O_4_ (cobalt iron oxide) and carbon (graphite). The phase ratios were calculated as 48.2% ± 0.7 CoFe_2_O_4_ and 51.8% ± 0.7 graphite, respectively. This distribution showed that the metal ferrite phase was successfully loaded onto the carbon matrix. The CoFe_2_O_4_ phase exhibited a typical cubic inverse spinel structure (*Fd*3̅*m*), and the refined lattice parameter *a* = *b* = *c* = 8.3920 ± 0.0014 Å. This value was consistent with the range of pure CoFe_2_O_4_ reported in the literature^20^ and indicated that the composite production process does not disrupt the spinel structure. The calculated unit cell volume was 591.441 Å^3^, which is consistent with normal spinel CoFe_2_O_4_ values.^20^ The microstructural parameter microstrain was 0.126% ± 0.007, indicating slight crystal lattice stresses resulting from the interaction of CoFe_2_O_4_ particles with the graphite matrix in the composite. The carbon phase, on the other hand, maintains its hexagonal graphite structure. The refined lattice parameters for graphite were *a* = *b* = 2.468 ± 0.002 Å and *c* = 6.7137 ± 0.0017 Å, which are consistent with the turbostratic graphite values in the literature. The unit cell volume was calculated as 35.432 Å^3^. The microstrain observed in the graphite phase was 0.13% ± 0.02, similar to the strain in the CoFe_2_O_4_ phase; this indicated that both phases in the composite were similarly affected by the synthesis conditions.

### 3.2 FT-IR Results

Figure 3 shows the FT-IR spectra of the samples. As a result of FT-IR analysis, the expected bond structures identified in the material include: OH groups, C–H bonds (from CH_2_ groups), C=O bonds, C=C bonds, C–OH bonds, C–O bonds, and metal–oxygen bonds such as Fe–O, Ni–O, and Co–O.^21,22^

**Figure 3:**
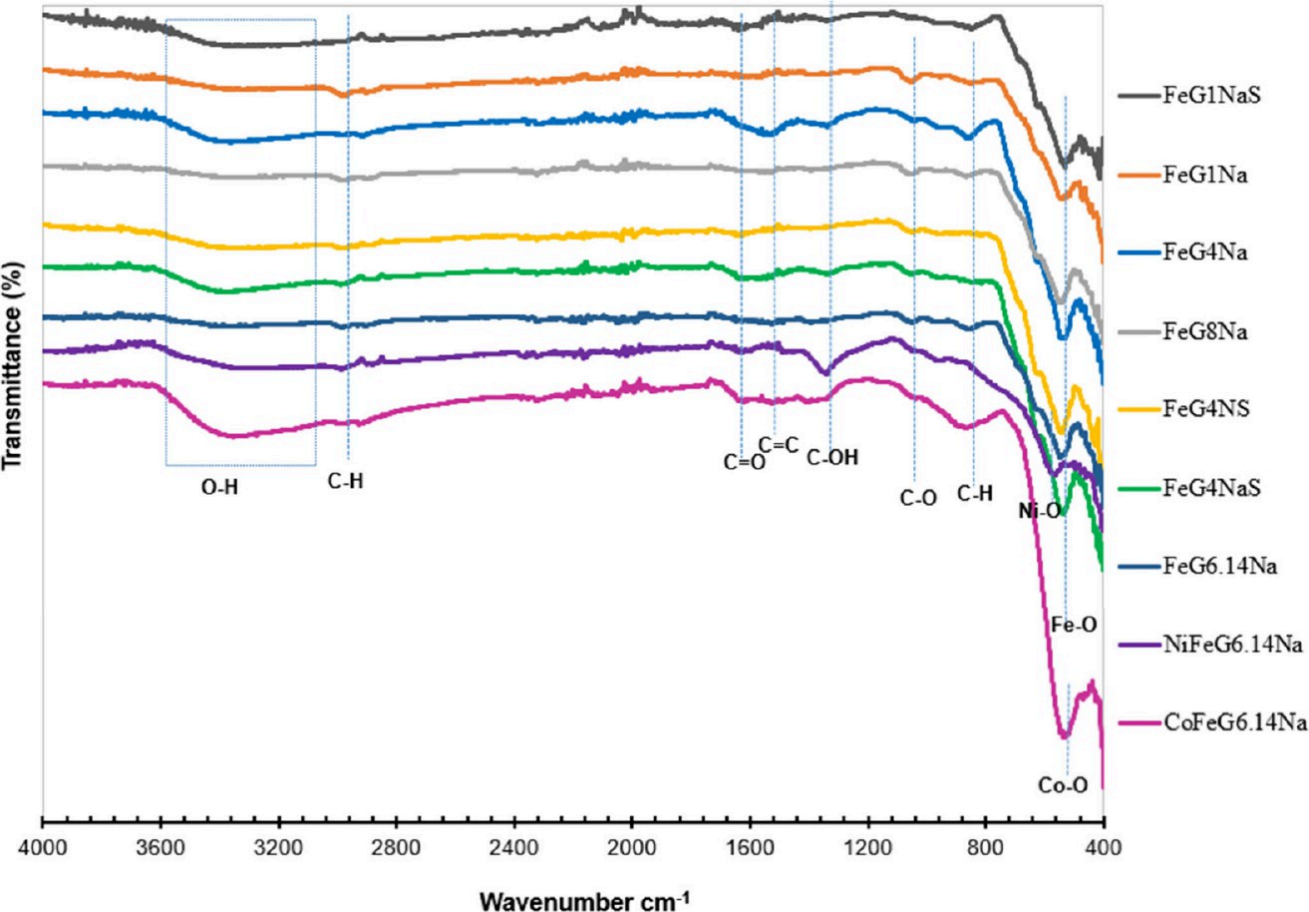
FT-IR spectra of the samples.

In FTIR spectroscopy, metal–oxygen (M–O) bonds typically appear in the range of 400–700 cm^–1^, and the exact position of the peak can shift depending on the metal’s atomic weight and bond strength. In the FT-IR spectra of the NiFeG6.14Na sample the peak at 570–575 cm^–1^ was related to Ni–O vibrations within the spinel ferrite composite, especially in the octahedral and tetrahedral sites. Literature sources confirm Ni–O presence in the ranges of 490–570 cm^–1^ and 575–640 cm^–1^.^19,23^ Similarly, a peak detected around 537 cm^–1^ was the indication of Co–O bonds in the CoFeG6.14Na sample.^24,25^ In all the samples with Fe, the peak at about 525–560 cm^–1^ region -especially around 530 cm^–1^ the sharp peak was attributed to the Fe–O stretching vibrations, which are typical for octahedral and tetrahedral sites in spinel structures.^26,27^ The metal bonds (Fe–O, Co–O, Ni–O) seen in the FT-IR graph indicate that they are bonded to the structure. Additionally, peaks near 840 cm^–1^ in the FT-IR spectrum of the samples were thought to result from C–H stretching vibrations.^21,28^ In samples synthesized using Fe^2+^/Fe^3+^, oxygen-containing groups increase on the graphite surface. The FT-IR results showed strong C–O stretching bands in the 1030–1050 cm^–1^ region, typically associated with epoxy groups. Previous studies report similar peaks at 1050 cm^–1^ and 1066 cm^–1^, indicating C–O vibrations.^29,30^ A noticeable C–OH stretching vibration is seen around 1360 cm^–1^, particularly in samples such as FeG1Na, FeG4Na, FeG6.14Na, FeG8Na, NiFeG6.14Na, and CoFeG6.14Na, which were synthesized using hydrated iron salts (FeCl_2_·4H_2_O and FeCl_3_·6H_2_O).^28,29^ The peak around 1550 cm^–1^ in the samples may indicate the presence of aromatic sp^2^-hybridized C=C bonds within the graphite structure.^31^ Samples such as FeG1Na, FeG4Na, FeG4NaS, and NiFeG6.14Na showed clear peaks at 1638 cm^–1^ range. This peak was related to the presence of carbonyl (C=O) groups. It was expected that such bonds could be formed due to the oxidation of graphite in the redox environment containing FeCl_2_ and FeCl_3_.^32^ The broad peaks around 3220–3225 cm^–1^ are observed in all samples, attributed to O–H stretching vibrations. Literature reported that the broad – OH bands typically appear around 3350 cm^–1^ and within the 3200–3400 cm^–1^ range, indicating the presence of hydroxyl groups.^28,33,34^

When comparing the FeG4NS and FeG4NaS samples, where all parameters were kept constant except for the pH adjusting agent (NaOH and NH_4_OH), it was observed that the FeG4NaS sample exhibited sharper spectral peaks. In particular, the – OH^–^ bond vibrations were more distinctly resolved. This enhanced clarity may be attributed to the use of NaOH as the pH-adjusting agent.

Another important point of consideration is the Fe–O vibrational peak, which may provide further insights into the coordination environment of iron within the sample. All the samples exhibited Fe–O peaks. However, the Fe–O peak of the samples (FeG1NaS, FeG4NS, FeG4NaS) prepared with FeCl_3_ and FeCl_2_ (anhydrous Fe salts) was sharper. This suggests that the syntheses made with anhydrous iron salt form purer and more crystalline Fe–O bonds. It has been stated in literature that the crystal structures obtained using anhydrous metal salts are more regular, stable, and controlled, unlike the hydrated forms.^35^

In the FeG6.14Na, NiFeG6.14Na, and CoFeG6.14Na samples, when the variation in the metal incorporated was considered as the sole variable, FT-IR analysis revealed that the Co-containing sample (CoFeG6.14Na) exhibited stronger C–H stretching peaks. This observation suggested a greater amount of organic residue on the surface. It was likely that Co^2+^ ions had interacted more strongly with organic compounds during the synthesis process, particularly at lower precipitation temperatures. In contrast, NiFeG6.14Na and FeG6.14Na showed weaker C–H signals, indicating the presence of less residual organic content.^36^ To compare the samples with different metal/C and Fe/C ratios, the results of FeG1NaS, FeG1Na, FeG4Na, FeG8Na, FeGI4Na, FeG4NS, FeG4NaS, FeG6.14Na, NiFeG6.14Na, and CoFeG6.14Na samples were examined. In the FeG8Na sample, the peaks corresponding to O–H, C=O, C=C, and C–O bonds appeared weaker as expected. This could be due to factors like reduced active surface area, agglomeration of metal oxides, surface passivation, and low dispersion. These factors may influence FT-IR peak intensities regardless of the chemical composition. In the C=O region of the FT-IR spectra, a very weak peak showed that only a small amount of carbonyl groups was present.

### 3.3 SEM and TEM Images

The surface morphologies of the synthesized samples were examined via SEM imaging (Figure 4). The effect of different metals (Fe, Ni–Fe, Co–Fe), metal salts (anhydrous or hydrated Fe salts), pH adjustment agents (NH_4_OH or NaOH), and metal/C mass ratio (*n:* 1, 4, 6.14, 8) on the morphological properties of magnetic nanoparticle–graphite composite were investigated. To evaluate the effect of the Fe/C ratio on the morphological properties of the materials, SEM images of FeG1Na, FeG4Na, FeG6.14Na, and FeG8Na samples were examined, and it was seen that the particle size increased as the Fe ratio increased. However, it was evaluated that this increase was not evident in the FeG8Na sample. When the SEM images of FeG4NaS and FeG4NS samples were examined to evaluate the effect of the pH adjustment agent (NH_4_OH and NaOH) on the morphological properties of the materials, it was seen that there was no significant morphological change when NH_4_OH was used. NaOH was preferred in the study for ease of use.

**Figure 4:**
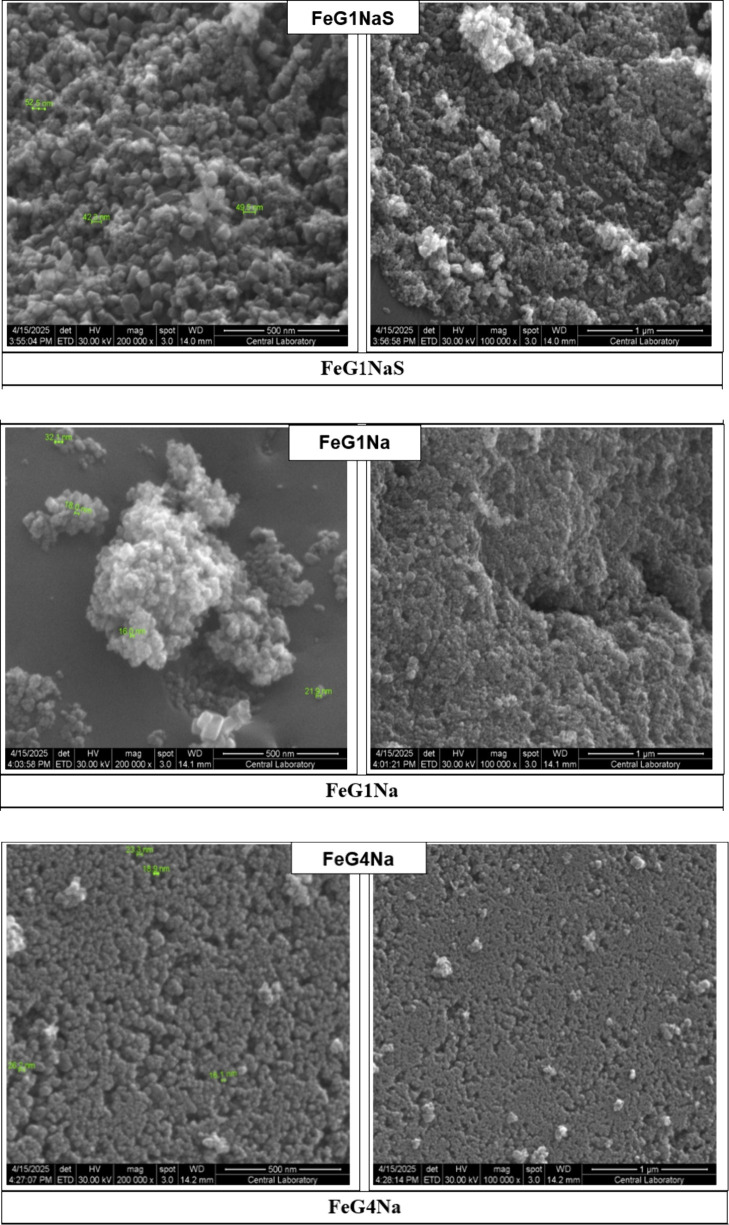

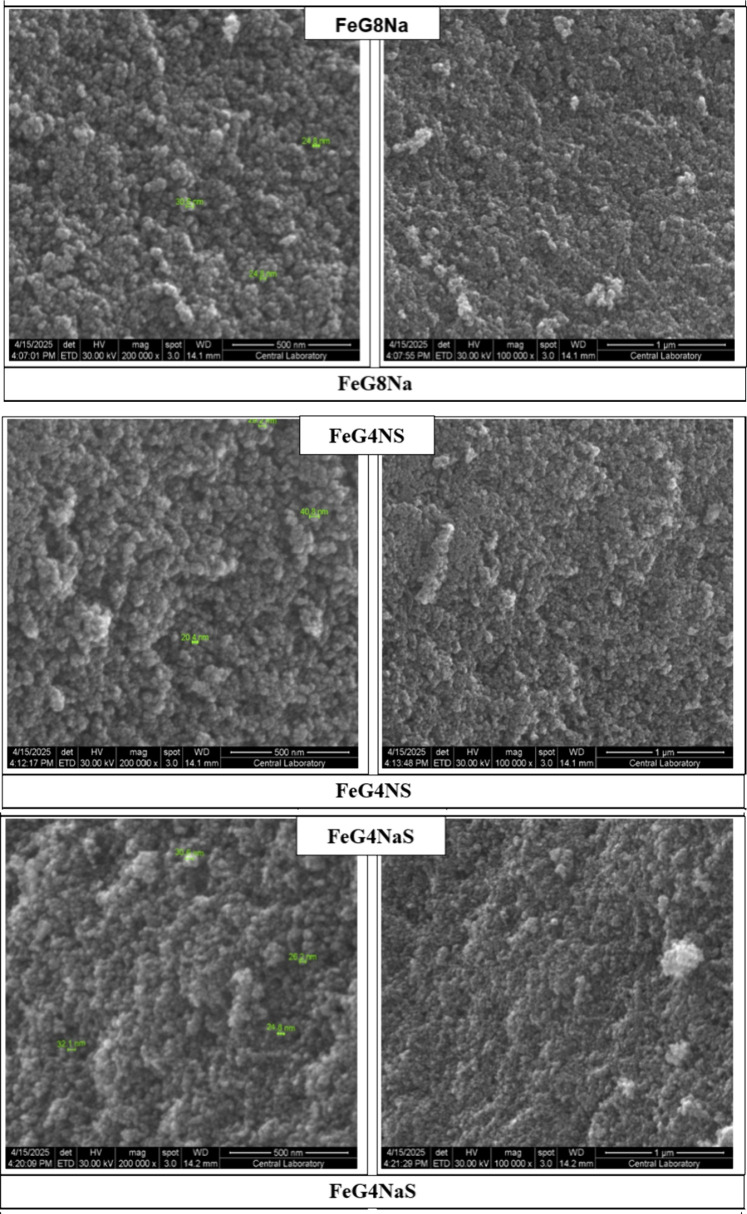

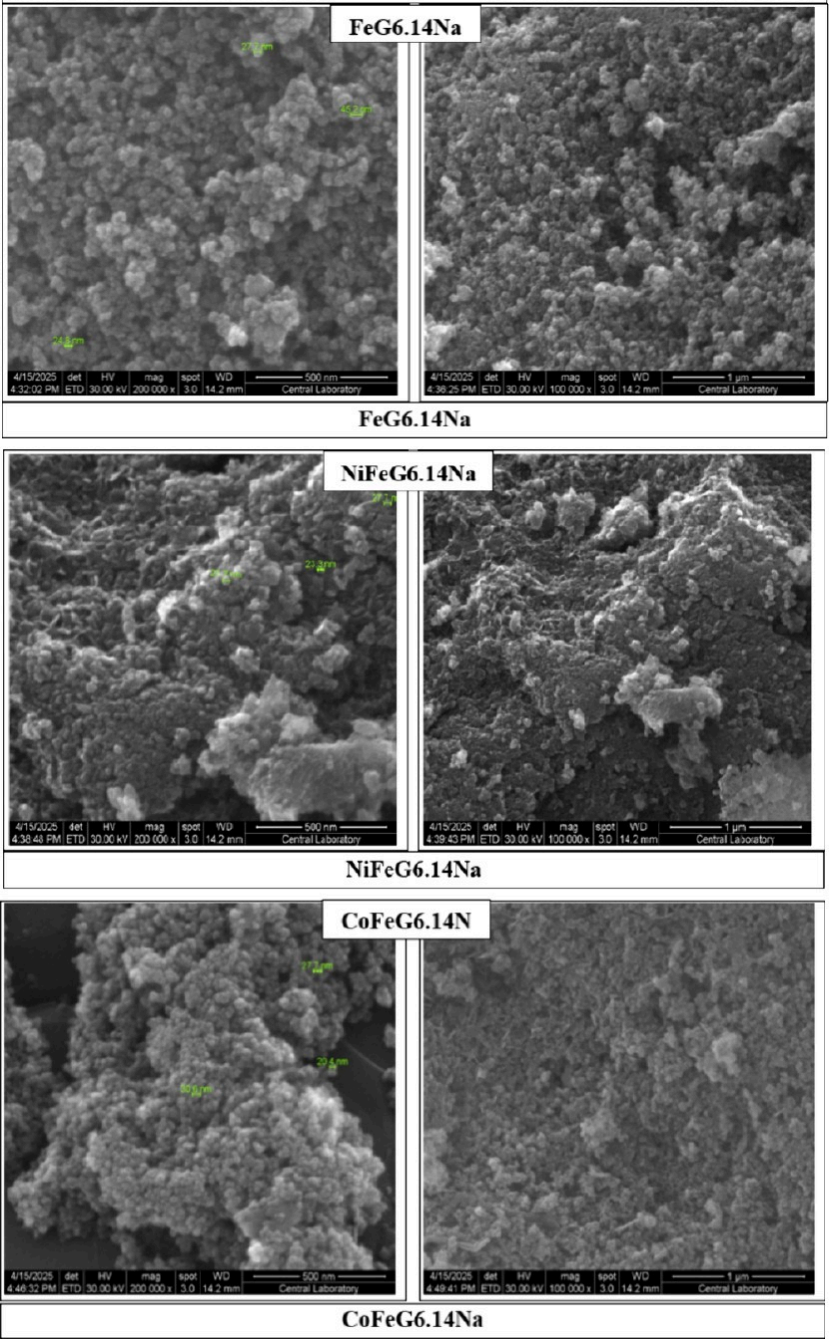
SEM images of the composite samples.

To evaluate the effect of anhydrous and hydrated Fe salt use, the SEM images of FeG1NaS and FeG1Na and FeG4NaS and FeG4Na samples were compared. As a result, it was seen that the use of anhydrous metal salt increased the particle size, and a more porous structure was obtained. When the SEM photographs of FeG6.14Na and NiFeG6.14Na samples were examined to evaluate the effect of different metal effects (Fe, Ni–Fe, Co–Fe) on the morphological properties of the materials, it was evaluated that agglomeration increased when Ni was added, but the average particle size did not change much. When the SEM photographs of FeG6.14Na and CoFeG6.14Na samples were compared, it was evaluated that the homogeneous structure was preserved when Co was added and there was no significant change in particle size and morphological structure.

The TEM images of FeG6.14Na samples are given in Figure 5. The presence of dark contrast regions corresponded to Fe_3_O_4_ nanoparticles, which were embedded and distributed within a lighter carbonaceous matrix. The lighter regions of the image were attributed to the graphite phase due to its lower electron density. The Fe_3_O_4_ nanoparticles exhibited partial agglomeration, a behavior that was typically associated with magnetic nanoparticles. The TEM results confirmed the successful formation of a Fe_3_O_4_-graphite hybrid composite structure. The results were confirmed with the literature.^37,38^ The SEM images of FeG6.14Na sample revealed the nanoparticles with sizes in the range of 24–45 nm, consistent with the TEM observations where Fe_3_O_4_ nanoparticles appear as dark contrast particles within the lighter graphite matrix. Both techniques confirmed the nanoscale nature of the composite, with SEM providing surface morphology and TEM giving detailed internal structure. Using the scale bar the interplanar distance was calculated as 0.255 nm. The obtained value of 0.255 nm is known to correspond to the characteristic crystal planes (311) of Fe_3_O_4_ in the spinel structure. This result confirmed that the sample is in the Fe_3_O_4_ phase, the crystal structure is well-ordered, and the lattice lines in the HRTEM image represented atomic planes specific to Fe_3_O_4_.

**Figure 5:**
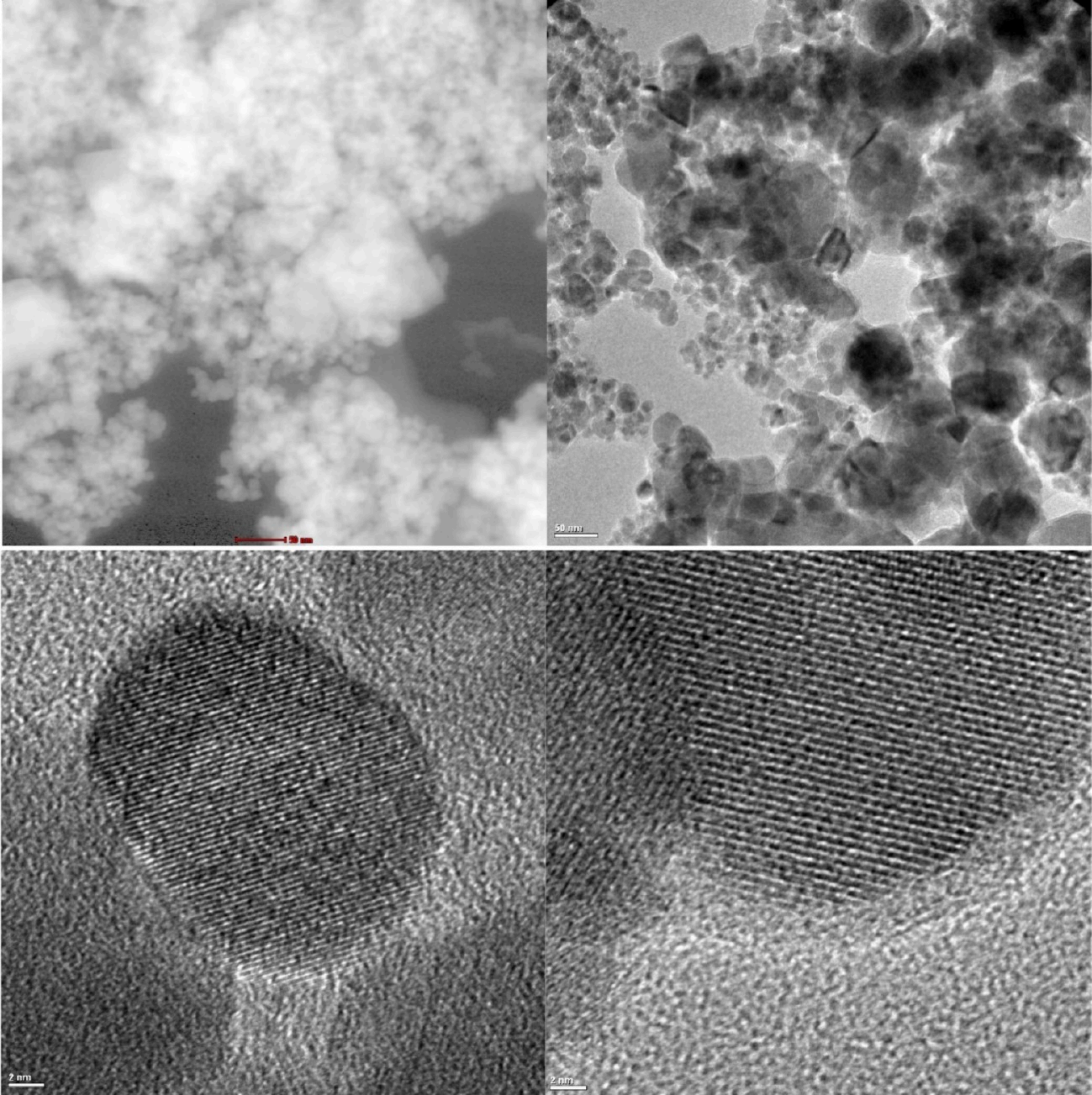
TEM images of the FeG6.14Na sample.

### 3.4 VSM Results

The magnetic properties of the synthesized FeG6.14Na, NiFeG6.14Na, and CoFeG6.14Na nanocomposites were investigated using Vibrating Sample Magnetometry (VSM) to evaluate their potential for electromagnetic interference (EMI) shielding and absorption applications (Figure 6).

**Figure 6:**
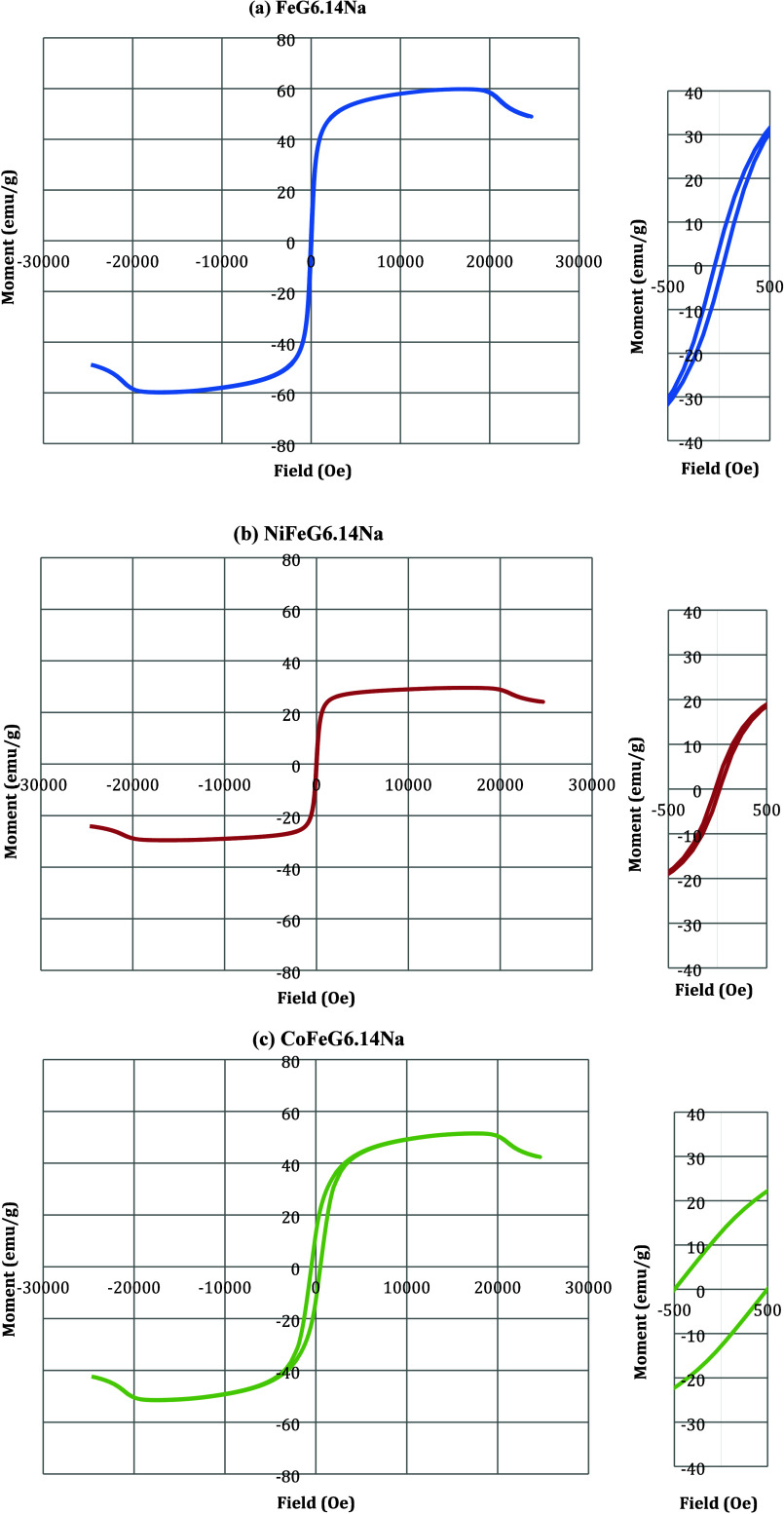
VSM hysteresis curves of the (a) FeG6.14Na, (b) NiFeG6.14Na, and (c) CoFeG6.14Na.

In the VSM analysis, saturation magnetization (*Ms*), remanence magnetization (*Mr*), and coercivity (*Hc*) values are important parameters (Table 2). Saturation magnetization, *Ms*, refers to the maximum magnetization a material can exhibit under an external magnetic field. It is seen that the FeG6.14Na sample exhibited the highest maximum magnetization. Remanent magnetization, *Mr, refers* to the residual magnetization retained by a ferromagnetic material after the external magnetic field has been removed. It is evident that this value is higher in the Co-containing material, which is also reflected in the broader hysteresis observed in the corresponding M–H curves. The *Hc* (coercivity) value is the amount of reverse magnetic field required to reduce the magnetization of a material to zero. Materials with a coercivity (*Hc*) value of less than 100 Oe are indicative of soft magnetic materials that can be easily magnetized and demagnetized with minimal energy loss, while *Hc* values between 100 and 1000 Oe indicate materials with moderate magnetic hardness and partial permanent magnetization ability. As can be seen, the CoFeG6.14Na sample showed semipermanent magnetization properties. It is well established in the literature that particle size and magnetization are closely correlated; smaller particle sizes typically result in lower magnetization due to increased spin arrangement disorder at the particle surface.^39^ This result was confirmed by the SEM image results of the FeG6.14Na, NiFeG6.14Na, and CoFeG6.14Na samples. As can be seen, the NiFeG6.14Na sample with a smaller particle size exhibited the lowest magnetic properties. As mentioned in the SEM results, NiFeG6.14Na exhibited a nonhomogenous structure. It is thought that the lowest magnetic behavior may also be related to the nonhomogeneous particle distribution of this material. Bracamonte et al. (2022) stated in their study that the saturation magnetization in graphite-based Ni nanocomposites was significantly affected by the distribution and crystallinity of magnetic nanoparticles.^40^ Similarly, in Fe_3_O_4_/RGO systems, Geng et al. (2021) reported that higher Fe_3_O_4_ loading increases *Ms* values up to 84 emu/g, but excessive Fe content leads to agglomeration and phase segregation, reducing the uniformity of magnetic response.^41^

**Table 2:** VSM Results of FeG6.14Na, NiFeG6.14Na, and CoFeG6.14Na Samples

Sample	*Ms*, emu/g	*Mr*, emu/g	*Hc*, Oe
FeG6.14Na	59.8	3.49	40.0
NiFeG6.14Na	29.6	1.45	21.7
CoFeG6.14Na	39.8	12.8	497.3

### 3.5 XPS Results

In the XPS analysis of the FeG6.14Na sample, the presence of Fe, O, and C was detected on the surface. The full spectrum revealed dominant signals corresponding to iron (Fe) and oxygen (O), as shown in Figure 7. The high-resolution O *1s* XPS spectrum of the FeG6.14Na sample indicated the presence of three chemically distinct oxygen species on the surface. The dominant peak at 529.91 eV (61.3%) corresponded to lattice oxygen (O^2–^), associated with Fe–O bonding in the oxide matrix. A second peak at 531.16 eV (36.3%) was attributed to hydroxyl groups (OH^–^), likely resulting from residual surface hydroxides or adsorbed-water due to the aqueous synthesis conditions. The weakest contribution, appearing at 533.96 eV (2.4%) was assigned to physically adsorbed molecular water (H_2_O). These findings indicated that the surface of FeG6.14Na consists of a mixed oxide/hydroxide structure, dominated by lattice oxygen, and are consistent with previously reported Fe_3_O_4_-based systems synthesized via coprecipitation methods.^42^ The high-resolution Fe *2p* XPS spectrum of the FeG6.14Na sample revealed the presence of both Fe^2+^ and Fe^3+^ oxidation states, indicating a mixed-valence iron environment. The spectrum was deconvoluted into multiple peaks with Fe *2p*_*3/2*_ peaks centered around 709.00 eV (Fe^2+^, 4.1%), 713.26 eV (Fe^2+^, 34.4%), and 716.19 eV (Fe^3+^, 26.8%). These values are in good agreement with those reported in the literature for Fe_3_O_4_ and mixed iron oxides.^43^ The Fe *2p*_*1/2*_ components appear near 721.90 eV (25.0%) and 726.34 eV (3.03%) were related to the Fe^2+^ and Fe^3+^ states, respectively. Notably, a prominent satellite peak at 732.34 eV and additional weaker satellite contributions such as at 729.26 eV are observed. These satellite features were the characteristics of Fe^3+^ species and further confirm the presence of trivalent iron. In addition, the satellite peak at about 719 eV was an indication of maghemite (Fe_3_O_4_) structure.^44^ Consequently, the Fe *2p* spectral analysis confirmed the coexistence of Fe^2+^ and Fe^3+^ species on the FeG6.14Na surface, indicative of a mixed-valence Fe_3_O_4_-like phase or a layered hydroxide/oxide hybrid structure. These results were in line with other XPS studies of iron oxides synthesized via coprecipitation methods.^42^

**Figure 7:**
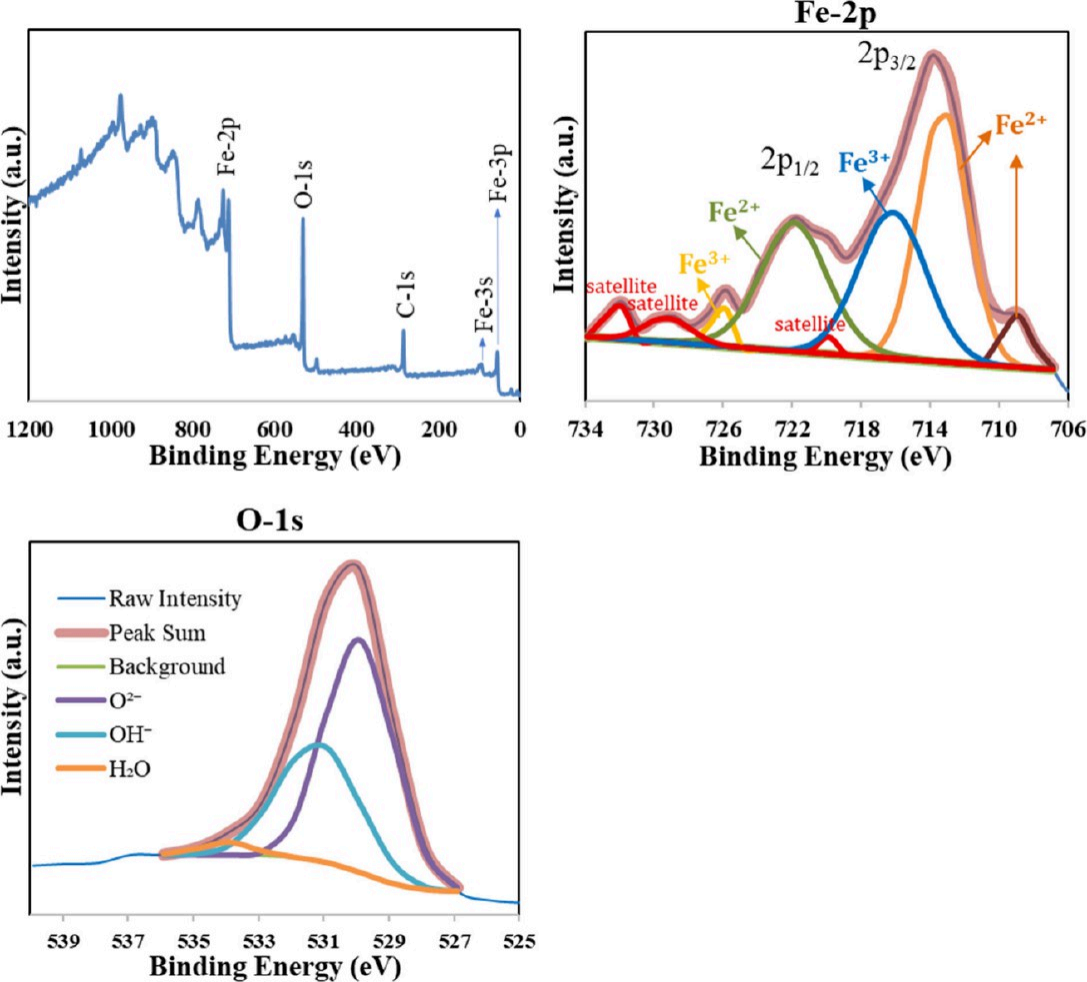
XPS spectra of FeG6.14Na sample.

In the XPS analysis of the NiFeG6.14Na sample, strong signals from Fe and O were detected on the surface, like the FeG6.14Na sample. Additionally, distinct peaks corresponding to nickel (Ni) were observed. In Figure 8 the high-resolution O *1s*, Fe *2p*, and Ni *2p* spectra of the NiFeG6.14Na sample and their interpretations are presented. The high-resolution Fe *2p* XPS spectra of the NiFeG6.14Na sample (Figure 8) revealed three major peaks, corresponding to characteristic features of iron in mixed-valence states. The main peak at 710.24 eV (50%) was attributed to the Fe *2p*_*3/2*_ core level of Fe^2+^, a common oxidation state in spinel-type ferrites such as NiFe_2_O_4_.^45^ A second peak at 723.65 eV (20.9%) was assigned to the Fe *2p*_*1/2*_ spin–orbit component, also corresponding to Fe^3+^. The third peak, located at 715.49 eV (29.1%) with a large fwhm (9.20 eV), was interpreted as a satellite feature, arising from multiple splitting due to unpaired *3d* electrons. This satellite is commonly observed in ferrite systems and indicates the presence of Fe^3+^ in a strongly correlated oxide environment.^45,46^ Overall, analysis of the Fe *2p* spectrum revealed that iron in the NiFeG6.14Na sample was predominantly present in the Fe^3+^ oxidation state, a feature commonly associated with spinel-type ferrites. The absence of Fe^2+^-specific peaks around ∼708 eV further supported this interpretation, suggesting complete oxidation or stabilization of Fe^3+^ within the NiFe_2_O_4_-like structure.

**Figure 8:**
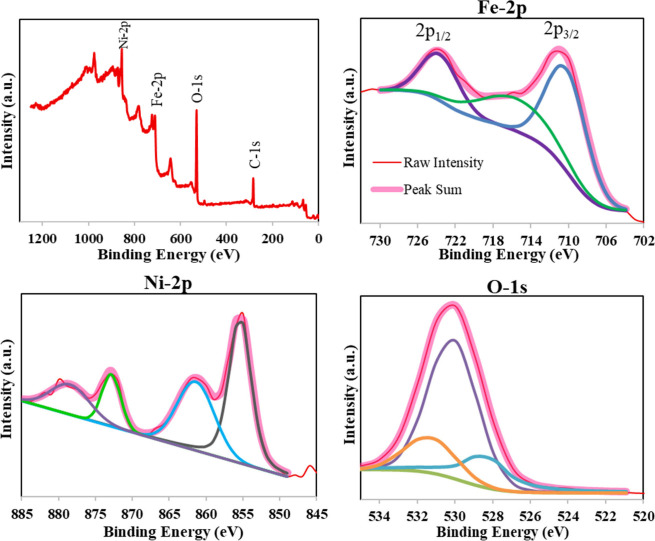
XPS spectra of NiFeG6.14Na sample.

The high-resolution Ni *2p* spectra of NiFeG6.14Na show four prominent peaks that are characteristic of Ni^2+^ species in spinel-type ferrite structures. The primary peak observed at 855.34 eV was attributed to the Ni *2p*_*3/2*_ component, indicating the presence of Ni^2+^ ions in octahedral coordination, which is characteristic of NiFe_2_O_4_-type spinel structures. This assignment agreed with previous findings reported by Hua et al. (2018).^45^ A strong satellite peak at 861.36 eV was also observed, which was typical for Ni^2+^ in oxides. Its high fwhm (5.95 eV) and distinct separation from the main peak confirmed the identity of the nickel as divalent and further ruled out metallic nickel or Ni^3+^ species. The Ni *2p*_*1/2*_ peak appeared at 872.83 eV, as expected for the spin–orbit split component of Ni^2+^. An additional satellite feature was detected at 878.44 eV, complementing the *2p*_*1/2*_ peak and confirming the high-spin Ni^2+^ state. Altogether, the presence of Ni *2p*_*3/2*_ at ∼855 eV, its associated satellite, and the *2p*_*1/2*_ doublet confirmed that Ni existed in the “+2” oxidation state in the NiFeG6.14Na sample. These results are consistent with spinel-type ferrite structures, where Ni^2+^ occupies octahedral sites, and match the reported XPS findings in both green-synthesized.^45^

The high-resolution O *1s* spectrum of NiFeG6.14Na (Figure 8) exhibits three deconvoluted peaks, each reflecting distinct oxygen chemical environments commonly observed in spinel ferrite systems and surface-bound species. The most intense peak at 530.11 eV was assigned to lattice oxygen (O^2–^) strongly bonded within the Ni–O and Fe–O framework of the spinel structure. This peak corresponded to metal–oxygen bonds in the crystalline bulk phase and confirmed the presence of well-formed ferrite lattices. This assignment aligned with values reported for NiFe_2_O_4_.^45,46^ The second peak at 528.41 eV was slightly shifted to lower binding energy and may represent under-coordinated surface oxygen or O^–^ species related to oxygen vacancies, defect sites, or loosely bound oxygen atoms on the surface. A third peak at 531.33 eV was attributed to surface hydroxyl groups (−OH) or chemisorbed oxygen-containing species such as carbonates or water molecules. These groups likely arise due to air exposure, moisture adsorption, or residuals from synthesis. This higher-energy oxygen species is often observed in oxide surfaces and is well documented in XPS studies on ferrites.^46^ The overall oxygen profile confirms that NiFeG6.14Na consists of a mixed oxygen surface with dominant contributions from lattice O^2–^, supported by defect-related and hydroxyl oxygen species, reflecting partial surface oxidation or hydration typical for ferrites synthesized via aqueous or hydrothermal routes.

Similar to the other samples, CoFeG6.14Na material, was synthesized through a coprecipitation method, potentially forming a Co­(II)–Fe­(III) layered hydroxide or partially oxidized structure. In the XPS analysis, signals corresponding to Co, Fe, and O were observed on the surface of CoFeG6.14Na. Below, the high-resolution spectra of Fe *2p*, Co *2p*, and O *1s* for the CoFeG6.14Na sample and their interpretations are presented (Figure 9).

**Figure 9:**
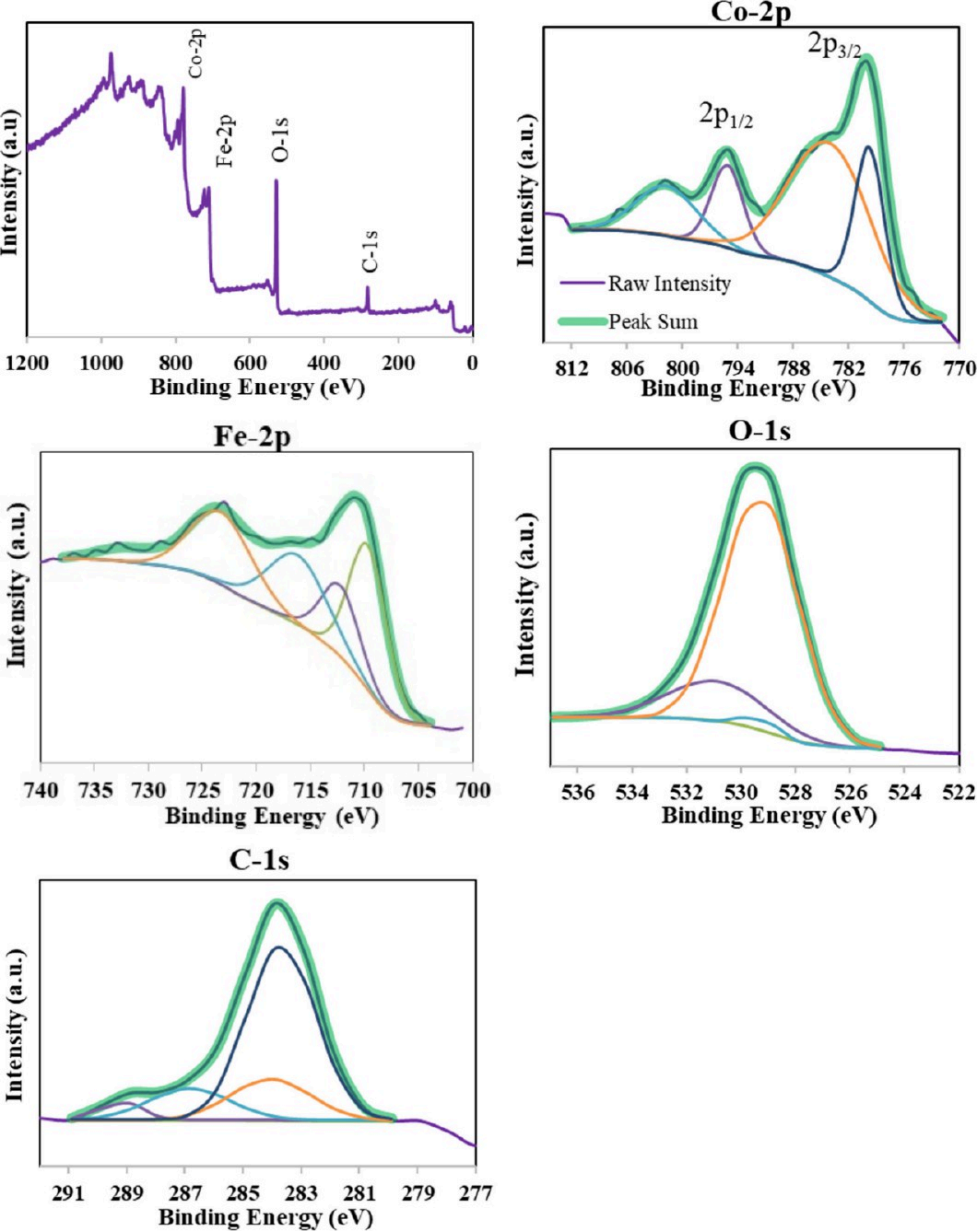
XPS spectra of the CoFeG6.14Na sample.

Figure 9 shows the three major peaks at 723.3 eV (25%) assigned to Fe *2p*_*1/2*_ and 709.7 eV (30%), 712.3 eV (17%) assigned to Fe *2p*_*3/2*_. These peaks suggested the presence of Fe^3+^ and Fe^2+^ species in the structure. The peak at 716.2 eV (24%) confirmed the existence of the Fe^3+^ oxidation state in the CoFeG6.14Na sample. The high binding energy shoulder at 731.8 eV (5%) was interpreted as a satellite peak, which was typical for Fe^3+^ state.^47,48^

The Co *2p* spectrum of CoFeG6.14Na (Figure 9) shows three major peaks characteristic of the cobalt oxidation state and coordination environment in spinel CoFe_2_O_4_-type structures. The most intense peaks at ∼784.02 eV (51.1%) and 779.7 eV (21.9%) corresponded to the Co *2p*_*3/2*_ core-level transition. This binding energy was typically attributed to Co^2+^ ions in tetrahedral and octahedral coordination.^47,49^ A smaller peak at 795.14 eV (12.5%) corresponded to the Co 2p_1/2_ spin–orbit component. These are consistent with standard spinel ferrite materials.^48^ Another satellite feature was observed at 801.76 eV (14.5%), which was often associated with shakeup transitions specific to Co^2+^ ions. The presence of this satellite peak confirmed the high-spin nature of Co^2+^ in the structure. These observations, including the binding energy positions, peak shapes, and satellite structure, are in strong agreement with the reported XPS characteristics of CoFe_2_O_4_.^48^ Thus, the XPS analysis confirmed the dominant oxidation state of cobalt in the CoFeG6.14Na sample to be Co^2+^, which was a typical signature of cobalt ferrite systems with octahedral site occupancy.

The O *1s* spectrum of CoFeG6.14Na (Figure 9) exhibits three distinct peaks, each representing different oxygen species associated with the material’s structure and surface chemistry. The most intense peak at 529.28 eV was attributed to lattice oxygen (O^2–^), indicating oxygen atoms bonded within the Co–O and Fe–O framework of the spinel structure. This binding energy is typical for metal–oxygen bonds in cobalt ferrites and is consistent with prior studies on CoFe_2_O_4_ nanoparticles.^48^ The second peak at 530.90 eV (Area: 7872.46) was ascribed to surface hydroxyl groups (−OH) or loosely bound oxygen species such as adsorbed water or oxygen-containing functional groups. The weaker component at 529.39 eV may represent either a satellite shoulder of lattice oxygen or oxygen in defective/vacant lattice sites. Its sharp and narrow profile indicated a relatively well-defined electronic environment. As a result, the spectral profile confirmed that the oxygen environment in CoFeG6.14Na was dominated by lattice-bound O^2–^, complemented by surface hydroxyl and possibly defect-related oxygen species.

The magnetization behavior of the FeG6.14Na, NiFeG6.14Na and CoFeG6.14Na composite samples correlated strongly with the cation distribution revealed by XPS. The FeG6.14Na sample exhibited the highest saturation magnetization (4.62 emu/g), which is consistent with its remarkably high Fe^2+^ content (64%). Since Fe^2+^ preferentially occupies the octahedral B sites, the increased B-sublattice moment strengthens the A-B superexchange interactions, resulting in a higher net magnetic moment. In the NiFeG6.14Na composite, the Fe^2+^/ Fe^3+^ ratio was nearly balanced (50%/49.9%), and Ni^2+^ strongly prefers B-site occupancy. This distribution produces a moderately inverted spinel structure with a lower B-site magnetic contribution than FeG6.14Na, which explains its lower *Ms* value (3.07 emu/g) and the lowest coercivity among the samples (21.7 Oe). The reduced coercive field was consistent with the low single-ion anisotropy of Ni^2+^. The CoFeG6.14Na sample contained the lowest Fe^2+^ fraction (17%), leading to a significantly weakened B-site magnetic moment and, therefore, a reduced *Ms* (3.08 emu/g). However, its coercivity was exceptionally high (497.3 Oe), which was attributed not to Fe^2+^/ Fe^3+^ distribution but to the presence of Co^2+^ ions. Co^2+^, known for its strong magnetocrystalline anisotropy and preferential B-site occupancy, greatly enhances domain-wall pinning, resulting in a large *Hc* despite the relatively low *Ms*.

### 3.6 UV–Vis DRS Measurements and Energy Band Gap Determination

UV–vis DRS results of the samples are given in Figure 10. The samples exhibited intense absorption in the 200–300 nm range and a broad absorption in the 300–800 nm range. The absorption band in the 200–300 nm range was attributed to π → π* transitions in the sp^2^ carbon structure, while the band observed in the 250–400 nm range originated from metal–oxygen ligand-to-metal charge transfer transitions; the visible region absorption in the 400–800 nm range was considered to be a combination of d-d transitions belonging to metal oxide phases, intervalence charge transfer, and band-tail states contributions. Comparing FeG1Na, FeG4Na, and FeG8Na samples, the prominence of the peak between 200 and 400 nm confirmed this assessment. The band gap values of the samples were calculated using UV–vis DRS results.

**Figure 10:**
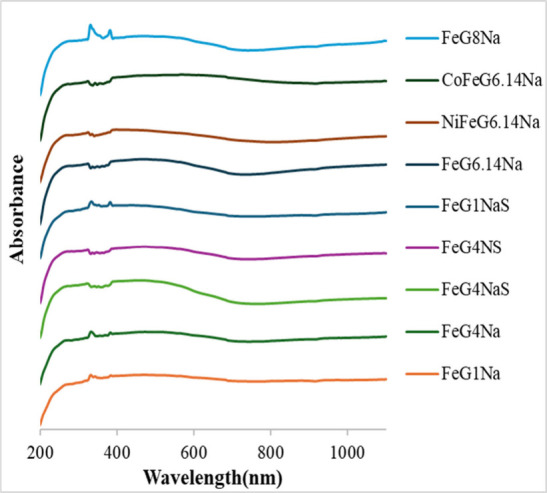
UV–vis DRS spectra of the samples.

The band gap is the minimum energy required to excite an electron from the top of the valence band to the trough of the conduction band. Once this minimum energy is reached, the sample can begin to absorb light, and electrons are excited from the valence band to the conduction band. The band gap energies (E_g_) of the samples were estimated by the Tauc formula^50^ (eq 1).

1 $${\left(\alpha h\upsilon \right)}^{n}=A\left(h\upsilon -E_{g}\right)$$

where A is a constant, *hυ* is the photon energy, E_g_ is the band gap, α is the absorption coefficient, n = 2 is the direct band gap for Fe_3_O_4_ nanoparticles. The band gap values of the samples were determined from the graph of (*αhυ*)^2^ versus *hυ* by extrapolating the linear region of the curve toward the photon energy axis where (*αhυ*)^2^ is equal to zero. The band gap values calculated for the samples are given in the Figure 11. According to previously published reports, the optical band gap energies of Fe_3_O_4_, NiFe_2_O_4_, and CoFe_2_O_4_ spinel ferrite phases typically lie within the range of 1.9–2.1 eV, which is consistent with their semiconducting nature and characteristic electronic transitions observed in UV–vis DRS analyze.^51,52^ The slightly lower values obtained here were thought to be due to the presence of graphite in the structure. While graphite, with its small band gap, allows for EMI transmission due to its high free electron density, metal oxides with a wider band gap provide EMI shielding dominated by absorption through electron transitions and magnetic/dielectric loss.

**Figure 11:**
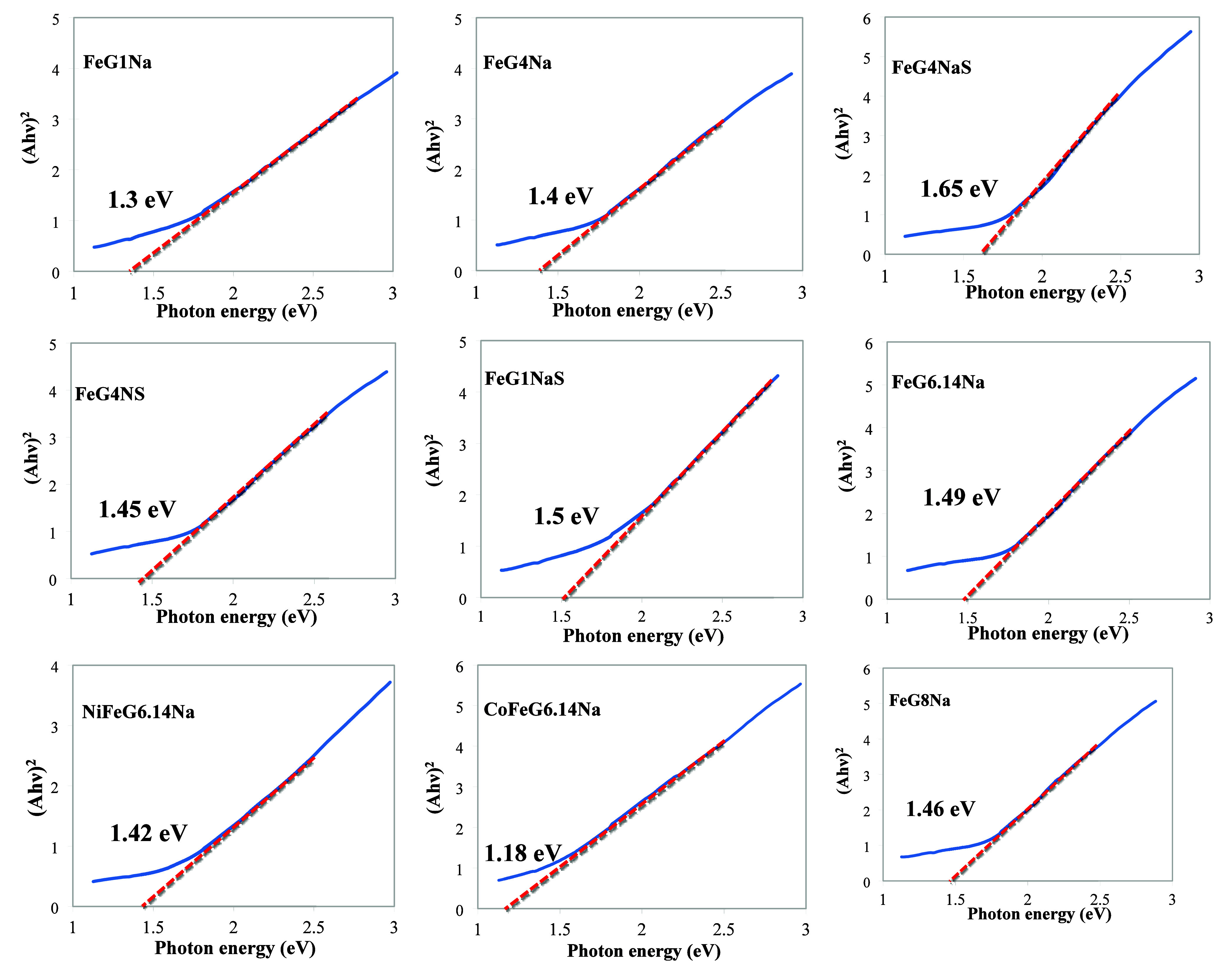
Tauc plot band gap energies of the samples.

### 3.7 EMI Shielding Performances

Electromagnetic wave absorption, as stated in the literature, refers to the part of the incident electromagnetic wave that is not lost through reflection and transmission and is calculated as follows.

Absorption(%)=100−Reflection(%)−Transmission(%)

This calculation is an important parameter of the EMI shielding quality of the material and shows how well the material absorbs or attenuates the electromagnetic wave.^3,5^ When the data in the table are evaluated based on the characterization findings and literature, the following relationships emerge.

The highest electromagnetic absorption result was obtained in the FeG1Na sample (19.6%) with a 40% contribution rate. This result is followed by FeG4Na (14.2%) and FeG4N (13.3%) samples with a high metal/carbon ratio. These results support the formation of pure and regular Fe-based spinel phases (Fe_3_O_4_) in XRD analysis (Figure 2). As stated in the literature, magnetic phases such as Fe_3_O_4_ strengthen the magnetic loss mechanism in the absorption of electromagnetic waves.^3,10^

The dense Fe–O bonds seen in FT-IR analyses (Figure 3) and the oxygen groups on the surface in XPS analyses (Figures 7, 8, and 9) indicate that metal oxides are in the formed structure in these samples. These results benefit the attenuation of electromagnetic waves by both electrical and magnetic losses.^8,46^ In particular, the oxygen groups and metal-oxide bonds on the surface support multiple electromagnetic scattering and absorption functions.^9^

In VSM analysis (Figure 6), it is seen that the FeG6.14Na sample has the highest magnetic saturation (Ms), while the electromagnetic wave absorption value (%2.5) is lower than the FeG1Na and FeG4Na, as seen in Table 2. This supports the fact that not only magnetic properties but also particle distribution, surface morphology, and graphite ratio are the conditions that affect the electromagnetic wave absorption performance.^40,41^ Indeed, in Ni and Co-doped samples (NiFeG6.14Na 1.6%; CoFeG6.14Na 1.8%), although the magnetic properties differed, the electromagnetic wave absorption values remained lower than the samples containing Fe. This can be interpreted as the Ni additive increasing agglomeration and disrupting homogeneity, while the Co additive imparts semipermanent magnetic properties but not being sufficient for optimum absorption, when looking at the characterization results.^45^

As a result, the electromagnetic wave absorption performance can be achieved by optimizing the crystal phase composition (Figure 2), particle size and distribution (Figure 4), surface chemistry (Figures 3, 7, 8, and 9), and magnetic properties (Figure 6) together. Table 3 data, consistent with the characterization findings and literature, reveal that Fe-based composites with optimum metal/carbon ratio and homogeneous structure exhibit the highest EMI absorption performance.^3–5^ Thus, it confirms once again that characterization studies are the key determinants in the design and improvement of electromagnetic performance.

**Table 3:** Surface Area Values of the Powders and EMI Shielding Performance of the Molded Samples

Sample	Multipoint Surface Area of the Powder (m^2^/g)	Additive (wt % to Paraffin)	Absorption Value (%)
Paraffin	-	-	0.8
Graphite	19.00	10	2.8
FeG1Na	32.03	40	19.6
FeG4Na	56.68	10	4.5
		20	6.3
		30	8.2
		40	14.2
FeG4NaS	70.02	10	3.0
FeG4NS	23.35	20	1.84
FeG4N	57.58	40	13.3
FeG6.14Na	54.46	10	2.5
NiFeG6.14Na	78.86	10	1.6
CoFe6.14Na	59.88	10	1.8
FeG8Na	76.62	40	5.4

## 4 Conclusion

In this study, the effects of different metals (Fe, Ni–Fe, and Co–Fe), metal salts (anhydrous or hydrated Fe salts), pH adjustment agent (NH_4_OH or NaOH), and metal/C mass ratio *(n*: 1, 4, 6.14, 8) on the properties of the magnetic nanoparticle–graphite hybrid composites and shielding efficiency were investigated. The cubic spinel structure of the FeG6.14Na, NiFeG6.14Na, and CoFeG6.14N samples were confirmed by XRD patterns. Crystallite sizes of the graphite phases in the FeG4Na, NiFeG6.14Na, and CoFeG6.14Na samples were calculated as 37.2, 34.3, and 28.1 nm, respectively. SEM images showed that particle size increased as the Fe ratio increased and the pH adjustment agent did not cause a significant change in the morphological structure. Additionally, the use of anhydrous metal salt increased the particle size, and a more porous structure was obtained. The TEM results confirmed the successful formation of a Fe_3_O_4_-graphite hybrid composite structure. VSM analysis revealed that the FeG6.14Na sample exhibited the highest saturation magnetization, whereas the NiFeG6.14Na sample with a smaller particle size demonstrated the lowest magnetic properties. When comparing the *Ms* values of FeG6.14Na, NiFeG6.14Na, and CoFe6.14Na samples, it was observed that FeG6.14Na > CoFe6.14Na > NiFeG6.14Na. As expected, the EMI shielding values also showed the same ranking. XPS results confirmed the coexistence of multiple oxidation states, including Fe^2+^, Fe^3+^, Ni^2+^, and Co^2+^, within the magnetic particles. In the electromagnetic interference (EMI) shielding performance tests, despite the variations in magnetic properties observed in the Ni- and Co-doped samples (NiFeG6.14Na: 1.57%; CoFeG6.14Na: 1.81%), their electromagnetic wave absorption capabilities remained inferior to those of the Fe-containing samples. When these results were evaluated in conjunction with complementary characterization analyses, it was inferred that Ni doping led to increased particle aggregation and a loss of homogeneity, whereas Co doping imparted semipermanent magnetic behavior that, although beneficial, was insufficient to achieve optimal absorption performance. Multiple point BET surface areas of the powder samples are given in Table 3. As the surface area increases, electron mobility increases, electromagnetic waves can be damped within the pore, and thus absorption occurs more efficiently. When comparing the EMI shielding results of FeG1Na, FeG4Na, and FeG8Na samples, it was observed that although the surface area value increased, the EM absorption value decreased as the Fe/C ratio increased. This indicates that the Fe/C ratio is a more dominant parameter. The band gap values of the samples were observed to range between 1.18 and 1.65, and no direct relationship was found between these values and EMI shielding behavior. As a result, characterization studies were evaluated as the key determinants in the design and improvement of electromagnetic performance.
